# Perceived attitudes of family and peers toward same-sex marriage as a distal sexual minority stressor for gay and bisexual men in Taiwan

**DOI:** 10.1186/s12889-022-14604-9

**Published:** 2022-11-21

**Authors:** Chih-Cheng Chang, Hsin-Yu Lu, Yu-Ping Chang, Ching-Shu Tsai, Cheng-Fang Yen

**Affiliations:** 1 Department of Psychiatry, Chi Mei Medical Center, and Department of Health Psychology, College of Health Sciences, Chang Jung Christian University, Tainan, Taiwan; 2 Department of Psychiatry, Kaohsiung Medical University Hospital, Kaohsiung, Taiwan; 3 School of Nursing, The State University of New York, University at Buffalo, New York, NY USA; 4 Department of Child and Adolescent Psychiatry, Chang Gung Memorial Hospital, Kaohsiung Medical Center, Kaohsiung and School of Medicine, Chang Gung University, 32 Dapi Rd. Niaosong Dist., Kaohsiung, 83341 Taiwan; 5 Department of Psychiatry, Kaohsiung Medical University Hospital, and School of Medicine College of Medicine, Kaohsiung Medical University, Kaohsiung; College of Professional Studies, National Pingtung University of Science and Technology, 100 Tzyou 1St Road, Kaohsiung, 80708 Taiwan

**Keywords:** Same-sex marriage, Psychological well-being, Gay, Bisexual, Stigma, Family, Peer

## Abstract

**Background:**

To investigate whether perceived attitudes of family and peers toward same-sex marriage (SSM) is a type of distal sexual minority stressor, as defined in minority stress theory, this cross-sectional study examined the associations of perceived attitudes of family and peers toward SSM with perceived sexual stigma from family and peers, internalized homonegativity, and mental health problems (e.g., depression, loneliness, anxiety) among gay and bisexual men in Taiwan.

**Methods:**

We recruited 400 gay and bisexual men and assessed their perceived attitudes of family and peers toward SSM; perceived sexual stigma from family and peers; internalized homonegativity; and severity of depression, loneliness, and anxiety.

**Results:**

Perceived attitudes of family and peers toward SSM (1) significantly correlated with various aspects of perceived sexual stigma from family and peers and (2) were significantly associated with internalized homonegativity, depression, loneliness, and anxiety.

**Conclusions:**

Perceived attitudes of family and peers toward SSM matched the characteristics of a distal sexual minority stressor, and as a new type of distal sexual minority stressor for lesbian, gay, and bisexual individuals, these perceived attitudes and related stress warrant greater attention from mental health professionals for the development of intervention programs.

## Background

Lesbian, gay, and bisexual (LGB) individuals have been reported to have a higher risk of mental health problems, such as depression [1, 2], anxiety [2], and loneliness [3], as well as suicide [1], compared with their heterosexual peers. Minority stress theory [4] is the most common theoretical framework used to explain mental health disparities between LGB individuals and heterosexuals. Sexual minority stress is defined as the unique stress experienced by LGB individuals living in a social environment characterized by heterosexualism and consequent prejudice and stigma toward LGB individuals [4]. Sexual minority stressors are broadly divided into distal stressors (e.g., victimization, discrimination, and microaggression against sexual minorities) and proximal stressors (e.g., internalized homonegativity, concealment of sexual orientation, and expectations of rejection) [4]. Research has indicated that sexual minority stressors compromise LGB individuals’ mental health directly or through the mediation of cognitive dysfunction (e.g., rumination) [4–6].

Perceived social attitudes toward LGB sexual orientation and culture are well recognized as a distal sexual minority stressor for LGB individuals [4]. Research has revealed that perceived unfavorable attitudes toward homosexuality from the public [7, 8] and family members and peers [9, 10] increase the risk of mental health problems among LGB individuals. As societies evolve, the unfavored targets of social attitudes rooted in heteronormativity may change and even expand. Legalization of same-sex marriage (SSM) has been intensely debated in recent decades [11–13]. As of June 20, 2022, SSM was legal in 29 countries [14]. Legalization of SSM not only extends equal rights to LGB individuals but also yields health benefits for this population [15–18]. However, unfavorable attitudes toward SSM remain prevalent in society, even in the countries or regions where SSM is legal [12, 19].

The campaign for SSM in Taiwan has been an arduous one. In May 2017, Taiwan’s Council of Grand Justices announced that the prevailing Civil Code that barred SSM was a violation of the human right to equality and was unconstitutional. However, people in Taiwan voted in a referendum to oppose changing the Civil Code to allow SSM. On the basis of the referendum results and the judgment of the Council of Grand Justices, the Taiwan government introduced a special act to legalize same-sex civil unions but not SSM for same-sex couples in May 2019 [20].

Whether perceived attitudes of family and peers toward SSM is a distal sexual minority stressor remains uncertain. According to minority stress theory [4], a distal sexual minority stressor should meet the following conditions: (1) it is specific to a sexual minority population; (2) it is strongly related to existing distal sexual minority stressors; (3) it contributes to the formation of proximal sexual minority stressors; and (4) it results in psychological distress. The stress triggered by perceived attitudes of family and peers toward SSM is specific to LGB individuals. Research has demonstrated that perceived unfavorable attitudes of family and peers toward SSM are associated with mental health problems among LGB individuals [9, 10]. However, perceived attitudes of family and peers toward SSM are not included in the currently used measures for sexual minority stressors (e.g., the 8-item Sexual Minority Stress Scale for Lesbian Women’s Everyday Lives [21], Couple-Level Minority Stress Scale [22], and Daily Sexual Minority Stressors Scale [23]) or sexual stigma (the Neilands Sexual Stigma Scale [24], Sexual Stigma Scale for Lesbian, Bisexual and Queer Women [25], and HIV- and Homosexuality-Related Stigma Scale [HHRS] [26]). Moreover, although research in the United States before the legalization of SSM nationwide indicated that LGB individuals living in states where SSM was banned experienced significantly higher levels of internalized homonegativity than those living in states where SSM was legal [27], the association of perceived attitudes of family and peers toward SSM with internalized homonegativity in LGB individuals has not been examined at the individual level. To elucidate the role of perceived attitudes of family and peers toward SSM as a distal sexual minority stressor, further study should examine its associations with the items of the existing scales measuring sexual stigma and internalized homonegativity.

In this cross-sectional study, we examined the associations of perceived attitudes of family and peers toward SSM with perceived sexual stigma from family and peers, internalized homonegativity, and mental health problems (depression, loneliness, and anxiety) among gay and bisexual men in Taiwan. We hypothesized that perceived attitudes of family and peers toward SSM (1) would have significant correlations with each item measuring the level of perceived sexual stigma from family and peers on the Homosexuality subscale of the HHRS, (2) would be significantly associated with internalized homonegativity, and (3) would be significantly associated with depression, loneliness, and anxiety.

## Methods

### Participants and procedure

We recruited participants by posting notices between August 2021 and January 2022 on several popular social media platforms (e.g., Facebook, LINE, and twitter), online message sharing applications (e.g., Bulletin Board System), and the websites of health promotion centers for LGB individuals. The inclusion criteria were as follows: (1) being aged ≥ 20 years; (2) self-identifying as a gay or bisexual man; and (3) residing in Taiwan. Individuals with conditions that might jeopardize their ability to comprehend the purpose of this study or the contents of the research questionnaires (e.g., having intellectual disability, brain injury, or cognitive impairment induced by alcohol or substance use) were excluded. Individuals interested in participating in this study were instructed to phone the research assistant, who ascertained their eligibility on the phone. Those who were eligible were invited for interviews at the psychiatric outpatient unit affiliated with a university hospital for completing the paper-and-pencil questionnaires. The research assistant was available to provide support as the participants completed the questionnaire. Four hundred gay or bisexual men provided written informed consent and participated in this study. This study was approved by the Institutional Review Board of Kaohsiung Medical University Hospital (KMUHIRB-F(I)-20,210,119).

### Measures

#### Perceived attitudes of family and peers toward SSM

We used one question in Mandarin to determine how participants perceived the attitudes of their family and peers toward SSM (“My family and peers did not accept same-sex marriage”) [10]. Participants rated the item on a 4-point Likert scale from 1 (*strongly disagree*) to 4 (*strongly agree*).

#### Mandarin Chinese version of the HIV- and homosexuality-related stigma scale

The self-administrated Mandarin Chinese version of the HHRS (MC-HHRS) is composed of two subscales measuring perceived stigma toward HIV and sexual minorities [26]. We used the 10-item Homosexuality subscale of the MC-HHRS (MC-HHRS-H) to assess perceived stigmatizing attitudes toward sexual minorities from families and peers among participants (e.g., “My family and peers unwillingly accept gay individuals” and “My family and peers would treat a gay individual differently than they would treat others”). Each item was rated on a 4-point Likert scale from 1 (*strongly disagree*) to 4 (*strongly agree*). The MC-HHRS was reported to have acceptable psychometric properties when administered to men who have sex with men in China [26]. The Cronbach’s α value of the Homosexuality subscale in the present study was 0.92.

#### Mandarin Chinese version of the measure of internalized sexual stigma for lesbians and gay men

We used the self-administered 17-item Mandarin Chinese version of the Measure of Internalized Sexual Stigma for Lesbians and Gay Men (MC-MISS-LG) to assess the level of internalized homonegativity in our sample [28, 29]. Each item was rated on a 5-point Likert scale from 1 (*strongly disagree*) to 5 (*strongly agree*). A higher MC-MISS-LG score indicates a higher level of internalized homonegativity. Both the original version [28] and Mandarin Chinese version [29] of the MISS-LG have satisfactory psychometric properties. The Cronbach’s α value of the MC-MISS-LG for this sample was 0.89.

#### Mandarin Chinese version of the center for epidemiological studies—depression scale

We used the 20-item Mandarin Chinese version of the Center for Epidemiological Studies—Depression Scale (MC-CES-D) to assess the severity of participants’ depression [30, 31]. All the MC-CES-D items were rated on a 4-point Likert scale from 0 (*rarely or none of the time*) to 3 (*most or all of the time*). A higher MC-CES-D score indicates more severe levels of depression. The MC-CES-D has been reported to have favorable reliability and validity [30, 32]. The Cronbach’s α value of the MC-MISS-LG for this sample was 0.92.

#### Mandarin Chinese version of the UCLA loneliness scale, version 3

We used the 20-item Mandarin Chinese version of the UCLA Loneliness Scale, Version 3 (MC-UCLALSV3) to assess participants’ feelings of loneliness [33, 34]. Participants rated each item on a 4-point Likert scale from 1 (*never*) to 4 (*always*). A higher MC-CES-D score indicates greater loneliness. The internal consistency and construct validity of the MC-UCLALSV3 have been reported to be satisfactory [33, 34]. The Cronbach’s α value of the MC-UCLALSV3 for this sample was 0.92.

#### Mandarin Chinese version of the state–trait anxiety inventory—state subscale

We used the 20-item the Mandarin Chinese version of the State–Trait Anxiety Inventory—State Subscale (MC-STAI-S) to assess participants’ level of anxiety. All items were assessed using a 4-point Likert scale from 1 (*almost never*) to 4 (*almost always*). A higher MC-STAI-S score indicates greater anxiety [35]. The MC-STAI-S was reported to be reliable and valid for a Taiwanese population [36]. The Cronbach’s α value of the MC-STAI-S for this sample was 0.95.

#### Demographic and sexual orientation factors

Data were collected on participants’ ages, education levels (high school or below vs. college or above), and sexual orientations (gay or bisexual).

### Data analysis

Descriptive statistics (mean, standard deviation [SD], and percentage) were first applied to summarize the participants’ characteristics, namely their demographics, sexual orientation, and scores on the measures (i.e., perceived attitude toward SSM from family and peers, the MC-HHRS-H, MC-MISS-LG, MC-CES-D, MC-UCLALSV3, and MC-STAI-S). The absolute skewness and kurtosis values for the scores of perceived attitudes of family and peers toward SSM, perceived sexual stigma from family and peers, internalized homonegativity, depression, loneliness, and anxiety ranged from 0.202 to 0.573 and 0.087 to 0.582, respectively; according to the guidelines of Kim [37], these scores were normally distributed.

We used three statistical models to examine the associations of perceived attitudes of family and peers toward SSM with perceived sexual stigma from family and peers, internalized homonegativity, and mental health problems. First, we used the intraclass correlation coefficient (ICC) to calculate the correlation between perceived attitudes of family and peers toward SSM and perceived sexual stigma from family and peers on each item of the MC-HHRS-H. ICC values of > 0.8, 0.6– 0.8, and < 0.6 indicate large, moderate, and small correlation sizes, respectively [38]. Second, we used multivariate linear regression analysis to examine the association of perceived attitudes of family and peers toward SSM (independent variable) with internalized homonegativity (dependent variable) as well as with depression, loneliness, and anxiety (dependent variable). Demographics and sexual orientation were covariates in the multivariate linear regression analyses. We also used criteria proposed by Baron and Kenny [39] to examine the moderating effects of demographics and sexual orientation on the associations of perceived attitudes of family and peers toward SSM with internalized homonegativity, depression, loneliness, and anxiety. A *p* value of < 0.05 was considered significant. All analyses were performed using IBM SPSS 20 (IBM, Armonk, NY, USA).

## Results

The 400 participants’ demographic information and data on perceived attitudes of family and peers toward SSM, perceived sexual stigma from family and peers, internalized homonegativity, and mental health problems are listed in Table 1. The mean age of the participants was 30.7 years (SD = 5.9 years); 83.2% had an education level of college or above; 83.2% self-identified as gay. The mean scores (SD) for perceived attitudes of family and peers toward SSM, perceived sexual stigma from family and peers, and the Social Discomfort, Sexuality, Identity subscales of internalized homonegativity were 3.1 (0.9), 26.9 (6.8), 19.0 (6.6), 11.3 (3.1), and 10.6 (4.5), respectively. The mean scores for the severity of depression, loneliness, and anxiety were 18.3 (11.1), 43.5 (11.1), and 39.2 (12.5), respectively.

**Table 1:** Sociodemographic Information and Data on Perceived Attitudes of Family and Peers Toward Same-Sex Marriage, Perceived Sexual Stigma, Integrated Homonegativity, and Mental Health Problems in the Sample (*N* = 400)

Variable	Mean (SD)	*n* (%)
Age (years)	30.7 (5.9)	
Education level		
Senior high school or below		44 (11.0)
College or above		356 (90.0)
Sexual orientation		
Gay		333 (83.3)
Bisexual		67 (16.8)
Perceived attitudes of family and peers against same-sex marriage	3.1 (0.9)	
Perceived sexual stigma from family and peers	26.9 (6.8)	
Internalized homonegativity		
Social discomfort	19.0 (6.6)	
Sexuality	11.3 (3.1)	
Identity	10.6 (4.5)	
Mental health problems		
Depression	18.3 (11.1)	
Loneliness	43.5 (11.1)	
Anxiety	39.2 (12.5)	

*SD*: Standard deviation

Table 2 presents the ICC results regarding the correlations of perceived attitudes of family and peers toward SSM with perceived sexual stigma from family and peers on each item of the MC-HHRS-H. The ICC values ranged from 0.635 to 0.826, indicating that perceived attitudes of family and peers toward SSM had a moderate to large correlation with the 10 items of perceived sexual stigma from family and peers on the MC-HHRS-H.

**Table 2:** Associations of Perceived Attitudes of Family and Peers Toward Same-Sex Marriage with Perceived Sexual Stigma from Family and Peers on the 10-Item Homosexuality Subscale of the HIV- and Homosexuality-Related Stigma Scale (HHRS)

Homosexuality subscale of the HHRS	Intraclass correlation coefficient
Item 1	0.764
Item 2	0.729
Item 3	0.635
Item 4	0.696
Item 5	0.826
Item 6	0.724
Item 7	0.640
Item 8	0.788
Item 9	0.653
Item 10	0.710

The multilinear regression results for the associations of perceived attitudes of family and peers toward SSM (1) with the Social Discomfort, Sexuality, and Identity subscales of internalized homonegativity on the MC-MISS-LG and (2) with depression, loneliness, and anxiety are presented in Tables 3 and 4, respectively. With the effects of age, education level, and sexual orientation controlled for, perceived attitudes of family and peers toward SSM was significantly associated with (1) increased internalized homonegativity on all dimensions of the MC-MISS-LG and (2) increased depression, loneliness, and anxiety severity.

**Table 3:** Associations of Perceived Attitudes of Family and Peers Toward Same-Sex Marriage with Internalized Homonegativity: Multivariate Linear Regression Analysis

	Internalized homonegativity					
	Social		Sexual		Identity	
	B (SE)	B (SE)	B (SE)	B (SE)	B (SE)	B (SE)
Age	0.150 (0.052)**	0.078 (0.065)	0.093 (0.025)***	-0.038 (0.028)	0.039 (0.036)	0.037 (0.036)
Education	0.613 (0.834)	0.584 (0.828)	0.194 (0.395)	0.177 (0.361)	0.235 (0.581)	0.213 (0.580)
Sexual orientation	2.985 (0.830)***	-0.750 (2.749)	2.063 (0.393)***	-0.125 (1.197)	2.992 (0.578)***	0.113 (1.807)
Perceived attitudes of family and peers against same-sex marriage	2.314 (0.353)***	2.248 (0.351)***	0.621 (0.167)***	0.515 (0.153)**	1.007 (0.246)***	0.996 (0.245)***
Interaction between age and perceived attitudes of family and peers against same-sex marriage		0.019 (0.011)		0.035 (0.005)***		
Interaction between sexual orientation and perceived attitudes of family and peers against same-sex marriage		1.093 (0.797)		0.599 (0.347)		0.867 (0.516)

*SB*: Standard error

^**^: *p* < 0.01

^***^: *p* < 0.001

**Table 4:** Associations of Perceived Attitudes of Family and Peers Toward Same-Sex Marriage with Depression, Loneliness, and Anxiety: Multivariate Linear Regression Analysis

	Depression	Loneliness		Anxiety
	B (SE)	B (SE)	B (SE)	B (SE)
Age	0.022 (0.092)	0.287 (0.091)**	0.200 (0.169)	0.071 (0.106)
Education	-2.816 (1.475)	-4.507 (1.448)**	0.320 (4.594)	-0.790 (1.694)
Sexual orientation	0.999 (1.468)	1.307 (1.442)	1.398 (1.445)	-0.720 (1.686)
Perceived attitudes of family and peers against same-sex marriage	2.877 (0.624)***	2.703 (0.613)***	2.727 (0.615)***	1.430 (0.717)*
Interaction between age and perceived attitudes of family and peers against same-sex marriage			0.027 (0.041)	
Interaction between education and perceived attitudes of family and peers against same-sex marriage			-1.515 (1.368)	

*SE*: Standard error

^*^: *p* < 0.05

^**^: *p* < 0.01

^***^: *p* < 0.001

Because that demographics and sexual orientation were significantly associated with some subscales of internalized homonegativity and mental health problems, the interactions between demographics, sexual orientation, and perceived attitudes of family and peers toward SSM were entered into multilinear regression analysis models to examine their associations with internalized homonegativity and mental health problems (Tables 3 and 4). The results indicated that the interaction between age and perceived attitudes of family and peers toward SSM was significantly associated with the Sexual subscale of internalized homonegativity, indicating that the association between perceived attitudes of family and peers toward SSM and the Sexual subscale of internalized homonegativity was stronger among older participants. Education and sexual orientation did not moderate the associations of perceived attitudes of family and peers toward SSM with internalized homonegativity and mental health problems.

## Discussion

Perceived attitudes of family and peers toward SSM were also significantly correlated with each item on the MC-HHRS-H. The MC-HHRS-H measures various aspects of perceived sexual stigma such as lack of acceptance of sexual minority or gender nonconformity, social barriers, familial disappointment, and stereotyping [26]. Perceived sexual stigma from family and peers is a typical distal sexual minority stressor. The significant correlation between perceived attitudes of family and peers toward SSM and perceived sexual stigma from family and peers highlights the role of perceived attitudes of family and peers toward SSM as a distal sexual minority stressor for gay and bisexual men. According to minority stress theory [4], multiple forms of distal minority stressors may successively or simultaneously contribute to the formation of proximal minority stressors and mental health problems. As a specific dimension of sexual minority stressor, perceived attitudes of family and peers toward SSM might be less influential in shaping internalized stigma and mental health problems than perceived general sexual stigma. However, as societies evolve, emerging sexual minority stressors such as perceived unfavorable attitudes of family and peers toward SSM warrant further study.

In this study, perceived attitudes of family and peers toward SSM was significantly associated with internalized homonegativity in gay and bisexual men. According to socioecological theory [40], internalized homonegativity is the result of the interaction between LGB individuals and their environments (e.g., microsystem, mesosystem, exosystem, and macrosystem). Family and peers are the microsystems that gay and bisexual men most frequently interact with. Research has revealed that perceived sexual stigma from family and peers contributes to the development of internalized homonegativity in LGB individuals [41, 42]. Considering that internalized homonegativity is one type of proximal sexual minority stressor, the significant association between perceived attitudes of family and peers toward SSM and internalized homonegativity underscores the role of perceived attitudes of family and peers toward SSM as a distal sexual minority stressor for gay and bisexual men. Moreover, the present study found that the association between perceived attitudes of family and peers toward SSM and the Sexual subscale of internalized homonegativity was stronger among older participants. Gay and bisexual men in Taiwan face the stress from the family obligations mandated in Confucianism to continue the family bloodline. Therefore, older gay and bisexual men may perceive greater attitudes of family and peers against SSM, feel stronger pressure, and develop greater internalized homonegativity regarding intimate gay relationships and sexual behaviors compared with younger gay and bisexual men.

Consistent with the results of previous studies [9, 10], we found that perceived attitudes of family and peers toward SSM was significantly associated with depression, loneliness, and anxiety in gay and bisexual men. Although mental health problems in gay and bisexual men have various etiologies [43], the impact of perceived attitudes of family and peers toward SSM on mental health in gay and bisexual men warrants consideration. For example, research has revealed that perceived attitudes of family and peers toward SSM might partially account for the high rates of suicidal ideation [44] and suicide attempts [45] among LGB individuals during the debate and referendum regarding SSM in Taiwan.

This study has some limitations. First, we used only one question to assess the attitudes of family and peers toward SSM; therefore, we could not identify the sources of attitudes toward SSM, the reasons for not accepting SSM, and the ways to perceive the attitudes. Given that all these characteristics of perceived attitudes of family and peers toward SSM may contribute to the sexual minority stress experienced by LGB individuals, further study is needed to deepen the understanding of perceived attitudes of family and peers toward SSM in LGB individuals. Second, because of the cross-sectional study design, we could not determine the temporal associations between perceived attitudes of family and peers toward SSM and internalized homonegativity and mental health problems. Third, the sample of this study was composed of gay and bisexual men with homogeneous age and education level; therefore, the findings may not be generalizable to all gay and bisexual men. Moreover, whether gay and bisexual men with various ethnicities, social classes, and urban and rural background perceive different levels of unfavorable attitudes of family and peers toward SSM also warrants further study. Fourth, we did not consider the participants’ gender identity; therefore, the interacting effect of sex and gender on perceived attitudes of family and peers toward SSM could not be determined.

The present study is the first one to examine the role of perceived attitudes of family and peers toward SSM as a distal sexual minority stressor. The results of this study did support the potential of perceived attitudes of family and peers towards SSM to be incorporated into a revised scale measuring sexual minority stressors. However, the contents and characteristics of perceived attitudes of family and peers toward SSM warrant further study. Furthermore, most of the items on the scales for sexual minority stressors (e.g., HHRS used in this study) measure perceived general sexual stigma but not the stigmatizing attitudes toward some specific characteristics of LGB individuals such as SSM. Therefore, how to integrate perceived attitudes of family and peers toward SSM into a revised scale measuring sexual minority stressors warrants further study.

## Conclusion

Perceived attitudes of family and peers toward SSM was significantly correlated with perceived sexual stigma from family and peers, internalized homonegativity, depression, loneliness, and anxiety in gay and bisexual men. Perceived attitudes of family and peers toward SSM matched the characteristics of a distal sexual minority stressor, as defined in minority stress theory. More research is needed on perceived attitudes of family and peers toward SSM and its associations with other distal minority stressors, proximal minority stressors, and mental health problems in LGB individuals. Mental health professionals should take perceived attitudes of family and peers toward SSM into consideration when developing intervention programs for mental health of LGB individuals.

## Data Availability

The data will be available upon reasonable request to the corresponding authors.
